# Supplementary material for: Telomerase RNA in Hymenoptera (Insecta) switched to plant/ciliate-like biogenesis
Source: Nucleic Acids Res. 2022 Dec 22;51(1):420–33. doi: 10.1093/nar/gkac1202 (PMC9841428; doi:10.1093/nar/gkac1202)
Supplement: gkac1202_Supplemental_Files [file gkac1202_supplemental_files.zip › Legends to Supplementary Tables.docx]

**Legends to Supplementary Tables**

**Table S1**: Summary of TR/telomere motif prediction: Species, in which we falied with TR/telomere motif predictions are highlited in red. Telomere motifs were predicted primarily based on TRFi analysis, corresponding TR template and previous studies (12, 13) - which exploited primarily chromsome level assembiles for telomere motif predictions. Conversely, independently prediceted TRs (inferred by Covartiance models - except Bombus TRs - in which telomere motif knowledge was utilised for initial filtering of TR-like candidates in geonome data) were checked for the presence of the type 3 snRNA promoter and correponding TR template region. Results in green - are in accordance with our predictions; NA - data were not available/analyzed; NP - data were analyzed, but we were not able to predict any relevant TR/telomere motif candidates.

**Table S2**: TRFi results from analysed WGS data are divided into separate spreadsheets according to Hymenoptera Families. The reslts are also accessible via corresponding hyperlinks below. Candidate telomere motifs are highlited in yellow. Species in which TRFi analysis did not provide candidate telomere motifs or which are not validated in TR template regions are in red. Results for each dataset are separated into 3 collums coresponding to 1 - tandem motif, 2 - complementary sequence, 3 - sum of reads containing particular tandem repeat. Corresponding SRA accession and species name is reffered in headline.

**Table S3**: Identification of type3 promoter snRNAs in available representative Arthropoda genomes (at NCBI) using Infernal tool with Covariance models available at RFAM for U1; U2; U3; U4; U5; U6; U6atac; MRP and 7SK RNAs. Identified sequences were extended with 200nts of adjacent genomic region ("Extended hit") to obtain their promoter regions. Significant extended hits were utilised for elucidation common/conserved type 3 promoter motifs using multiple sequence alignments and motif searches (e.g. MEME suite).

**Table S4**: Identification of TR-like homologs by Infernal tool across Lepidoptera and Trichoptera representatvive genomes at NCBI. Primary Lepidopteran TR sequences were predicted de novo in genomes of Plutela xyllostela and Spodoptera exigua as a sequences contatining type 3 promoter + template-like region and showing homology in other related Lepidoptera species. Proposed TR homologs ("Infernal hit") were extended with 200 nt genomic context to obtain their promoter regions ("Extended hit").

**Table S5**: Summary of the most frequent repeats found at the assembly termini of Leptopillina boulardi (GenBank accession number: GCA_019393585.1) genome. The repeats were identified de novo and annotated to the genome using pipeline RepeatExplorer2 (TAREAN included) with the short Illumina pair-end raw reads available in NCBI SRA (GenBank accession number: SRR11665922) as an input.

**Table S6:** Oligonucleotides
